# A Genital Infection-Attenuated Chlamydia muridarum Mutant Infects the Gastrointestinal Tract and Protects against Genital Tract Challenge

**DOI:** 10.1128/mBio.02770-20

**Published:** 2020-11-03

**Authors:** Sandra G. Morrison, Amanda M. Giebel, Evelyn Toh, Arkaprabha Banerjee, David E. Nelson, Richard P. Morrison

**Affiliations:** a Department of Microbiology & Immunology, University of Arkansas for Medical Sciences, Little Rock, Arkansas, USA; b Department of Microbiology & Immunology, Indiana University School of Medicine, Indianapolis, Indiana, USA; University of Nebraska Medical Center

**Keywords:** *Chlamydia*, animal models, genital tract immunity, intracellular pathogen, pathogenesis, virulence

## Abstract

*Chlamydia* spp. productively infect mucosal epithelial cells of multiple anatomical sites, including the conjunctiva, lungs, gastrointestinal (GI) tract, and urogenital tract. We, and others, previously established that chlamydial GI tropism is mediated by distinct chromosomal and plasmid factors. In this study, we describe a genital infection-attenuated Chlamydia muridarum mutant (GIAM-1) that is profoundly and specifically attenuated in the murine genital tract. GIAM-1 infected the murine GI tract similarly to wild-type (WT) Chlamydia muridarum but did not productively infect the lower genital tract of female mice, ascend to infect the upper genital tract, or cause hydrosalpinx. However, GI infection of mice with GIAM-1 elicited a transmucosal immune response that protected against subsequent genital challenge with WT Chlamydia muridarum. Collectively, our results demonstrate that chlamydia mutants that are profoundly attenuated for specific organ tissues can be derived and demonstrate that live-attenuated vaccine strains that infect the GI tract, but do not elicit genital tract disease, could be used to protect against chlamydia genital tract infection and disease.

## Abstract (precis)

Chlamydia is the most common sexually transmitted bacterial infection in the United States. Most chlamydia genital infections resolve without serious consequences; however, untreated infection in women can cause pelvic inflammatory disease and infertility. Antibiotics are very effective in treating chlamydia, but most genital infections in both men and women are asymptomatic and go undiagnosed. Therefore, there is a critical need for an effective vaccine. In this work, we show that a mutant chlamydia strain, having substantially reduced virulence for genital infection, colonizes the gastrointestinal tract and produces robust immunity to genital challenge with fully virulent wild-type chlamydia. These results are an important advance in understanding chlamydial virulence and provide compelling evidence that safe and effective live-attenuated chlamydia vaccines may be feasible.

## INTRODUCTION

Chlamydia trachomatis (Ct) urogenital infection is the most common bacterial sexually transmitted infection (STI) in the United States, with 1.8 million cases reported in 2018, a 19% increase since 2014 (1). In women, Ct infection can spread from the cervix into the uterus and fallopian tubes, causing inflammation and serious reproductive complications. In contrast, urethral Ct infections in men usually elicit local inflammation and self-limiting urethritis symptoms (2) and only rarely cause upper genital tract complications (3). Ct rectal infections are also prevalent in both sexes and are often detected in individuals who do not report high-risk behaviors that are risk factors for other rectal STIs (4, 5). Ct urogenital infections are susceptible to first-line antibiotics (6), but several factors including the high prevalence of asymptomatic Ct urogenital infections (7), limited immunity conferred by prior infections (8), and lower antibiotic sensitivity of rectal Ct infections (9, 10) suggest that a vaccine will be needed to decrease Ct prevalence rates (11, 12).

The ability of several veterinary *Chlamydia* spp. to colonize the gastrointestinal (GI) tracts of animals for years without causing overt pathology or disease is well documented (13). Similarly, Ct is frequently detected in rectal specimens from women and men who have sex with men who report no GI symptoms (5, 14–16). Although the natural history of rectal Ct infection in humans is unclear, nonhuman primates inoculated rectally with Ct shed infectious organisms and develop no signs of proctitis (17–19). Mice have been used extensively to model the natural history of rectal *Chlamydia* infections. When mice are challenged with Chlamydia muridarum (Cm) by either oral gavage or direct rectal inoculation, they often remain infected indefinitely without developing GI pathology (10, 20).

It has been proposed that *Chlamydia* spp. encode an array of niche-specific virulence factors (21). For example, urogenital-tropic Ct strains encode functional tryptophan synthase genes, whereas these genes are inactivated or missing in the ocular-tropic Ct trachoma strains (22, 23). Even more subtle genetic polymorphisms may explain the differing virulence of Ct trachoma isolates in nonhuman primates (24). Confirmation of the roles of putative Ct tissue-tropism genes in humans has been difficult due to obvious ethical constraints, but recent studies in mice confirm that some Cm virulence factors play disproportionate roles in specific tissues. For example, the Zhong lab demonstrated that a Cm strain transformed with a plasmid that lacks *pgp3* colonizes the lower murine genital tract but is unable to cause upper genital tract disease or disseminate to or colonize the GI tract (25). They further show that chromosomal genes *tc0237* and/or *tc0668* are linked to Cm genital tract virulence and GI tract colonization (26, 27). In our recent studies, we isolated two Cm mutants that infected the mouse genital tract and caused upper genital tract disease but were unable to colonize the GI tract following either oral gavage or rectal inoculation (28). Both mutants have a nonsense mutation in Cm *tc0600*, an ortholog of Ct *ct326* previously linked to Ct GI tropism in humans (29), but also share other background mutations.

Since mutations in both the chlamydial plasmid and chromosome can alter Cm GI tropism in mice, we wondered if other mutations could affect Cm genital tropism. Here, we describe a Cm mutant (GIAM-1) that infects the murine GI tract but is highly compromised in its ability to infect the murine genital tract and induce genital tract disease. Despite the inability of GIAM-1 to cause genital tract disease, rectal inoculation of mice with this mutant elicited transmucosal immune responses that protected mice against subsequent wild-type (WT) Cm genital infection and associated pathology. The specific mutation(s) that mediates GIAM-1 genital attenuation was not identified, but our results suggest that specific chromosomal genes also play disproportionate roles in Cm genital tropism and support the hypothesis that chlamydiae employ an array of tissue-specific virulence factors. Importantly, targeted disruption of genital tropism genes could yield live-attenuated vaccines that are incapable of eliciting clinical disease but are able to prime protective immunity.

## RESULTS

### Phenotypic and genomic characteristics of GIAM-1.

We previously isolated a mutant from a heavily ethyl methanesulfonate (EMS)-mutagenized Cm library that formed small inclusions during the early to mid-stage of the developmental cycle (12 to 24 h postinfection) (30). This mutant, initially referred to as delayed-development “dd” mutant, was renamed GIAM-1. Results of one-step *in vitro* growth curve assays demonstrated that GIAM-1 had a 42-fold-lower recovery of inclusion-forming units (IFU) compared to WT Cm at 18 h postinfection (hpi), but this decreased to less than a 2-fold difference in IFU by 30 h postinfection. Genome sequencing of GIAM-1 revealed a total of 58 mutations including 26 missense and 6 nonsense mutations (Table 1).

**TABLE 1:** Summary of missense and nonsense SNPs in GIAM-1^*a*^

Mutation position	Old locus tag	Nucleotide substitution	Amino acid substitution	Gene ID	Description
795	TC0001	G-A	Arg-Gln	TC_RS00005	Porphobilinogen synthase HemB
30370	TC0023	C-T	Ala-Thr	TC_RS00120	LPS export ABC transporter ATP-binding protein
32915	TC0027	M-G	Leu/Ile-Val	TC_RS00140	Hypothetical protein
78526	TC0068	C-T	Arg-Trp	TC_RS00360	Hypothetical protein
80447	TC0070	C-T	Cys-Tyr	TC_RS00370	tRNA uridine-5-carboxymethylaminomethyl(34) synthesis GTPase MnmE
99394	TC0083	C-T	Pro-Ser	TC_RS00440	Phosphoenolpyruvate carboxykinase (GTP)
119133	TC0100	C-T	Asp-Asn	TC_RS00525	Cadmium-translocating P-type ATPase
147536	TC0122	C-T	Glu-Lys	TC_RS00650	Oxygen-independent coproporphyrinogen III oxidase
174699	TC0143	C-T	Pro-Ser	TC_RS00755	Bifunctional UDP-N-acetylmuramate–l-alanine ligase/d-alanine–d-alanine ligase
212252	TC0178	C-A	Gln-His	TC_RS00915	Glycine–tRNA ligase (glyQS)
**308945**	**TC0262**	**G-A**	**Gln-STOP**	**TC_RS01330**	**Autotransporter domain-containing protein–pmpE/F2**
**368944**	**TC0312**	**C-T**	**Trp-STOP**	**TC_RS01565**	**Glycogen-debranching protein (like glgX)**
**473585**	**TC0412**	**C-T**	**Gln-STOP**	**TC_RS02045**	**Hypothetical protein**
**537553**	**TC0440**	**C-T**	**Gln-STOP**	**TC_RS02195**	**DUF1669 domain-containing protein–putative PLD**
621109	TC0513	C-T	Ala-Val	TC_RS02605	OmpH family outer membrane protein
649908	TC0539	C-T	Asp-Asn	TC_RS02735	N-acetylmuramoyl-l-alanine amidase
712192	TC0595	C-T	Gly-Arg	TC_RS03010	Preprotein translocase subunit SecE
715323	TC0598	G-A	Gly-Glu	TC_RS03035	Hypothetical protein
719578	TC0602	C-T	Asp-Asn	TC_RS03055	DEAD/DEAH box helicase
747521	TC0623	G-A	Ala-Thr	TC_RS03155	Lon protease
750533	TC0626	C-T	Ser-Phe	TC_RS03170	Tyrosine recombinase XerC
783341	TC0655	C-T	Glu-Lys	TC_RS03320	Malate dehydrogenase
819206	TC0686	G-A	Gly-Glu	TC_RS03480	Transcriptional repressor NrdR
852272	TC0714	C-T	Cys-Tyr	TC_RS03610	Diaminopimelate epimerase
890544	TC0746	C-T	Pro-Ser	TC_RS03775	UDP-2,3-diacylglucosamine diphosphatase LpxG
904731	TC0762	G-A	Gly-Arg	TC_RS03875	Methylated-DNA–[protein]-cysteine S-methyltransferase
973257	TC0839	C-T	Thr-Ile	TC_RS04265	d-Alanyl-d-alanine carboxypeptidase
**975526**	**TC0841**	**C-T**	**Gln-STOP**	**TC_RS04275**	**RsmB/NOP family class I SAM-dependent RNA methyltransferase**
980329	TC0843	C-T	Pro-Ser	TC_RS04285	DEAD/DEAH box helicase
**1000611**	**TC0864**	**G-A**	**Gln-STOP**	**TC_RS04385**	**DNA mismatch repair endonuclease MutL**
1029460	TC0884	C-T	Val-Ile	TC_RS04485	Thio:disulfide interchange protein
1053812	TC0909	G-A	Thr-Ile	TC_RS04620	Hypothetical protein

a Synonymous and intergenic mutations are not listed. Nonsense mutations are highlighted in bold.

### GIAM-1 is significantly attenuated in a murine genital tract infection model.

**FIG 1:**
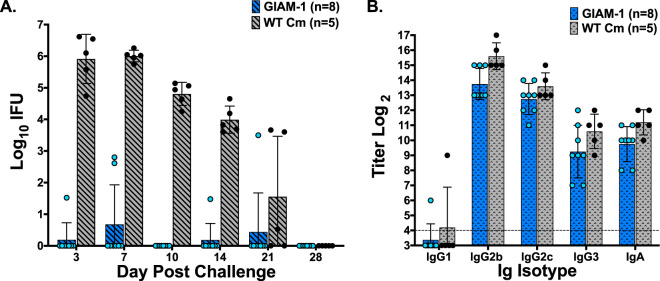
GIAM-1 infection is highly attenuated in the murine genital tract. Female mice were treated with medroxyprogesterone acetate and challenged vaginally with 5 × 10^4^ IFU of either GIAM-1 or WT Cm. Infection was monitored by collecting vaginal-cervical swabs at the indicated time points and enumerating IFU (A). IFU shedding was greatly reduced in the GIAM-1-challenged mice compared to WT-infected mice throughout the course of infection (*P* < 0.0001, days 3, 7, 10, and 14; *P* > 0.05, days 21 and 28). GIAM-1 infection also did not result in genital tract pathology at 72 days postinfection: 0 of 8 (0%) GIAM-1-infected mice developed hydrosalpinx, whereas 5 of 7 (71%) WT Cm-infected mice were hydrosalpinx positive. Even though GIAM-1 was highly attenuated for genital infection, serum antibody responses were similar in mice challenged with WT Cm or GIAM-1 at day 49 postinfection (*P* > 0.05) (B). Dashed horizontal line represents the starting dilution for serological analysis (1/16).

To determine if the delay in *in vitro* inclusion maturation that we observed for GIAM-1 impacted *in vivo* virulence, mice were vaginally challenged with GIAM-1 or WT Cm, and the course of infection was followed by enumerating IFU recovered from vaginal/cervical swabs collected at various time points postinoculation. GIAM-1 exhibited marked attenuation for genital infection compared to WT Cm (Fig. 1A). The majority of mice challenged with GIAM-1 did not develop discernible genital tract infection, and those that developed infection shed 2 to 3 log_10_ fewer chlamydiae. Although GIAM-1 displays a minor growth defect in cell culture, infectivity of this mutant is profoundly attenuated in the murine genital tract. Furthermore, genital tract pathology, as assessed by the development of hydrosalpinx, was highly attenuated in mice challenged with GIAM-1. Zero of 8 GIAM-1-challenged mice developed hydrosalpinx (0%), whereas 4 of 5 (80%) mice challenged with WT Cm were hydrosalpinx positive.

Forty-nine days following vaginal challenge, mice were bled and sera were analyzed by ELISA for antichlamydia antibodies (Fig. 1B). All mice, irrespective of the challenge Cm strain, developed robust antibody responses to Cm. Thus, although mice challenged with GIAM-1 failed to establish a productive genital infection, they developed systemic antichlamydia antibody responses, suggesting that GIAM-1 was infecting another anatomical site.

### GIAM-1 infects the lower GI tract and induces transmucosal protective immunity to WT Cm genital challenge.

**FIG 2:**
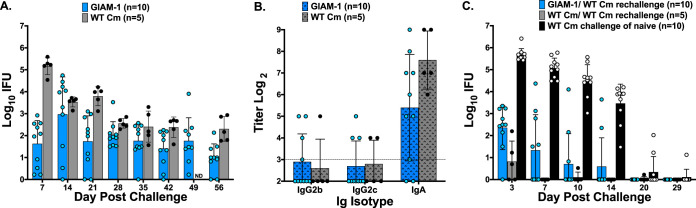
GIAM-1 infects the GI tract, induces mucosal anti-Cm antibodies, and protects against a WT Cm genital infection. Mice were inoculated rectally with 1 × 10^5^ IFU of either GIAM-1 (*n* = 10) or WT Cm (*n* = 5). Rectal swabs were collected weekly for 8 weeks, and IFU were enumerated (A) (*P* < 0.0001, day 7; *P* < 0.01, day 21; *P* > 0.05, days 14, 28, 35, 42, and 56). GIAM-1 versus WT. Mice were then treated with doxycycline for 10 days, rested for 2 weeks, and were culture negative for chlamydiae prior to vaginal wash collection and the rechallenge experiment. Vaginal washes were collected on day 76 post-primary rectal infection from the mice represented in panel A, and antichlamydia antibody responses were measured (B). Dashed horizontal line represents the starting dilution for vaginal wash antibody analysis (1/8). Antibody responses did not significantly differ between WT Cm- and GIAM-1-infected mice (*P* > 0.05). Mice in panel A and a group of naive mice were treated with medroxyprogesterone acetate and then challenged vaginally 5 days later with 5 × 10^4^ IFU of WT Cm (C). GIAM-1 rectally infected mice shed >3 log_10_ fewer IFU than did age-equivalent naive mice (*P* < 0.0001, days 3, 7, 10, and 14), and the shedding of chlamydiae did not differ significantly from that of mice that were infected rectally with WT Cm (*P* > 0.05). ND, not determined.

GI infections established either by the direct inoculation of Cm into the GI tract or by the dissemination of Cm from the genital tract to the GI tract are characterized by the continuous shedding of infectious chlamydiae from the GI tract in the absence of GI pathology (28, 31, 32). Because vaginal inoculation of GIAM-1 elicited a robust antibody response in the absence of a severely attenuated genital tract infection, we tested if GIAM-1 could infect the GI tract. Mice were challenged rectally with either WT Cm or GIAM-1. Rectal inoculation of mice with either WT Cm or GIAM-1 resulted in rectal shedding of infectious chlamydiae for at least 56 days, the entire duration of the experiment (Fig. 2A). In general, GIAM-1-infected mice shed fewer IFU than WT Cm-infected mice, but all mice were culture positive at one or more sampling time points. Additionally, cross-colonization of the genital tract was not detected in any of the WT Cm or GIAM-1 rectally inoculated mice, corroborating prior observations (33, 34).

At 76 days post-rectal infection, vaginal washes were collected from WT Cm- and GIAM-1-infected mice and mucosal anti-Cm antibody responses were measured. WT Cm and GIAM-1 rectally infected mice produced comparable titers of vaginal wash anti-Cm IgG_2b_, IgG_2c_, and IgA, which confirmed that the ability of GIAM-1 to elicit transmucosal antibody responses was not compromised (Fig. 2B). To determine if rectal infection with GIAM-1 conferred protective immunity to genital challenge, the infected mice were treated with doxycycline, resolution of the GI infection was confirmed by culture, and mice were then challenged vaginally with WT Cm (Fig. 2C). Compared to naive mice challenged vaginally with WT Cm, both rectally WT Cm- and GIAM-1-infected mice showed marked protection against genital challenge. Protection was characterized by a 3 to 4 log_10_ reduction in vaginal/cervical IFU shedding and a substantially shortened course of infection. At 9 weeks following vaginal rechallenge with WT Cm, mice were evaluated for the development of hydrosalpinx. Seven of 10 (70%) naive (nonimmune) mice developed hydrosalpinx following vaginal WT Cm challenge, whereas only 3 of 10 (30%) of the GIAM-1 rectally infected mice and 0 of 5 (0%) WT Cm rectally infected mice developed hydrosalpinx. Thus, GIAM-1 rectal infection elicits transmucosal protective genital tract immunity, albeit somewhat less robust than that elicited by WT Cm rectal infection.

### GIAM-1 translocates from the genital tract to establish GI tract infection.

**FIG 3:**
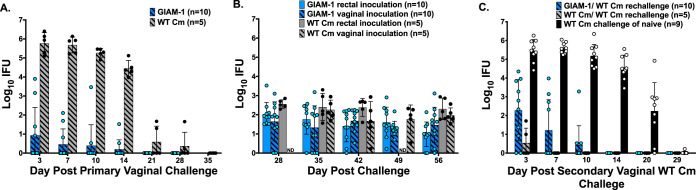
Vaginal inoculation of GIAM-1 results in GI tract infection that subsequently protects against WT Cm vaginal challenge. Medroxyprogesterone acetate-treated mice were challenged vaginally with 5 × 10^4^ IFU of either GIAM-1 or WT Cm. The course of infection was followed by collecting vaginal swabs and enumerating IFU at the indicated time points (A) (*P* > 0.0001, days 3, 7, 10, and 14). At 28 days post-vaginal challenge, and weekly thereafter, mice were swabbed rectally to determine if the GI tract had become infected as the result of vaginal challenge. Data from the two groups of naive mice that were directly inoculated rectally with either GIAM-1 or WT Cm from Fig. 2A are included for comparison (B). Similar levels of GI infection were observed in all groups of mice (*P* > 0.05 for all comparisons). Sixty days following primary genital challenge with either WT or GIAM-1, mice were cured of infection with doxycycline and rechallenged with 5 × 10^4^ IFU of WT Cm (C). Primary vaginal inoculation with GIAM-1 conferred significant protective immunity to rechallenge with WT Cm (GIAM-1 versus naive, *P* < 0.0001), and the protection conferred was nearly as robust as a primary vaginal WT Cm inoculation (*P* < 0.05, day 3, and not significant, *P* > 0.05, at the other time points). Age-matched naive mice inoculated vaginally with WT Cm are shown for comparison.

GIAM-1 is strikingly attenuated for genital tract infection, yet vaginally infected mice produce a robust anti-Cm antibody response (Fig. 1), suggesting possible colonization of another anatomical site. Naive mice inoculated vaginally with WT Cm normally develop a self-limiting genital infection that resolves in approximately 4 weeks but also acquire long-term Cm GI tract colonization (20, 35). To determine if GIAM-1 retained the ability to disseminate to the GI tract, we tested whether vaginal inoculation with GIAM-1 resulted in GI infection. As previously observed (Fig. 1A), GIAM-1 was highly attenuated for genital tract infection (Fig. 3A). Beginning at day 28 post-vaginal infection, a time when genital infection had resolved, and continuing weekly thereafter, rectal swabs were collected and the number of recoverable IFU was compared to mice rectally inoculated with either WT Cm or GIAM-1 (Fig. 3B). Although the shedding of infectious chlamydiae as detected by rectal culture varied among the animals, all of the mice had at least one positive rectal culture. Furthermore, the number of IFU recovered from rectal swabs of mice challenged vaginally with either WT Cm or GIAM-1 did not significantly differ from that of mice challenged rectally with either WT Cm or GIAM-1, respectively (*P* > 0.05). Thus, WT Cm and GIAM-1 exhibited similar abilities to autoinoculate the GI tract following vaginal inoculation.

To determine if immunity developed in mice that were vaginally inoculated with GIAM-1 and which subsequently established GI infection, mice were cured of GI infection by doxycycline treatment and were then challenged vaginally with WT Cm (Fig. 3C). Although immunity to genital tract reinfection, as assessed by IFU shedding and duration of infection, was more robust in mice that had received a primary WT Cm challenge, primary genital infection with GIAM-1 produced substantial immunity to WT Cm rechallenge. Notably, only 1 of 10 (10%) GIAM-1 vaginally infected–WT Cm-rechallenged mice developed hydrosalpinx, whereas hydrosalpinx was observed in 4 of 5 (80%) of the WT Cm vaginally infected–WT Cm-rechallenged mice.

## DISCUSSION

A chlamydia vaccine is needed because existing STI control measures have been ineffective at curbing the rise in infection rates (1). Although most chlamydia STIs are self-limiting, adverse infection outcomes are a significant cause of reproductive morbidity in both men and women. Current chlamydia vaccine research has focused heavily on the development of subunit and recombinant vaccines, but when tested using the murine infection model, none have yet achieved the “gold standard” level of protection that is elicited by prior chlamydia infection. How natural immunity develops to genital chlamydia in humans is unclear because, unlike the GI tract, the female reproductive tract lacks highly organized lymphatic tissues. The role of GI tract infections in the development of genital tract immunity to chlamydia has not been evaluated in humans, but infection of the murine GI tract with Cm provides robust transmucosal protection against genital Cm infection (28). These observations have spurred renewed interest in the potential of live-attenuated chlamydia vaccines. Supporting this possibility, Kari et al. showed that nonhuman primates vaccinated with an attenuated Ct trachoma isolate were partially protected against ocular reinfection and disease when inoculated with a virulent trachoma isolate (36). Identification of an attenuated Ct strain that colonized the GI tract without causing overt disease and which was unable to infect urogenital tissues would be an ideal candidate for further vaccine studies. Thus, the goals of this study were to determine if Cm encodes genital tract-specific virulence factors and, if so, to test if corresponding Cm mutants that do not cause genital tract disease, but do infect the GI tract, induce protective immunity to virulent Cm genital tract challenge. Overall, our results show that tissue-specific virulence factors contribute to chlamydia genital tract pathogenesis *in vivo* and that inactivation of tropism factors can yield effective attenuated vaccine strains.

Studies examining the natural history and consequences of human GI chlamydia are just now beginning to emerge, and therefore, it is difficult to ascertain the significance of our findings in relation to human GI infection. However, similarly to Cm GI infections in mice, Ct GI infections in humans are cleared slowly, yield few symptoms, and elicit little or no pathology (37–39), and the lower GI tract of humans can also be infected with Ct via direct inoculation (40). Like many experimental models of human disease, there are limitations that temper extrapolation of our results using the mouse model of GI infection to humans. First, it is unknown if Ct, like Cm, disseminates from the genital tract to the GI tract via an internal route (31), and Cm GI infections do not autoinoculate the genital tract in female mice (34). Second, only limited evidence supports the hypothesis that oral inoculation causes Ct GI infections in humans (4, 41), whereas this is well established in the Cm mouse model (13, 28). Finally, it is unknown if Ct GI infections induce transmucosal genital tract protective immune responses in humans. Answering these questions is challenging because concomitant rectal and genital Ct infections are prevalent in humans (42), and the order and routes by which these infections are acquired are usually difficult to discern. Incomplete understanding of how and if autoinoculation, dissemination, and sexual behaviors contribute to the establishment of GI infections is also a significant barrier. Considering that naturally acquired rectal Ct infections are susceptible to common antibiotics (43), and the power of GI induction of transmucosal genital tract protection in the mouse model is well established (44), infectious challenge studies in male human volunteers may now be warranted to clarify the potential routes by which Ct GI infections can be acquired, the natural history of GI infections, and whether GI infections trigger antichlamydia immune responses that could protect against genital infection.

Attenuated vaccines have reduced virulence and yet retain the ability to infect the host and elicit protective immunity. Similarly to other attenuated vaccines that colonize the GI tract and protect against disease (e.g., polio vaccine), GIAM-1 colonizes the GI tract and protects against the serious pathological consequences that often follow chlamydia genital infection. The specific immunological mechanism(s) of the transmucosal immunity elicited by GIAM-1 GI infection is not known, but robust mucosal IgA is elicited and may be key to the immune protection. Unlike in the pregenetics era when attenuated vaccines were developed by serial passage of virulent organisms and mechanisms of attenuation were unknown, simple tools for inactivation of specific genes in Ct chlamydia strains now exist (45), and more sophisticated genetic engineering tools developed for other Ct biovars could be adapted for this purpose (46–48).

The goal of our study was to identify a Cm mutant that exhibited striking genital tract infection attenuation without impacting GI tract virulence. We screened a subset of heavily EMS-mutagenized Cm isolates using the murine GI and genital tract models of infection and identified a mutant, GIAM-1, that exhibited those characteristics. Unfortunately, the large number of nonsense mutations in GIAM-1 and the mutant’s lack of a strong counterselectable *in vitro* phenotype have prevented us from identifying the attenuating mutations using markerless lateral gene transfer and/or complementation (49, 50). Nonetheless, although we have screened only a small number of heavily mutagenized Cm isolates in the GI and genital tract infection models, those screens have identified the genital tract infection-attenuated GIAM-1 mutant and other mutants that are specifically attenuated for GI infection (28). Thus, our results suggest that chlamydiae encode many genes that play disproportionate roles in specific tissues. Since a wide range of potential tissue tropism phenotypes can be evaluated in Cm mouse models, this hypothesis should be increasingly testable as the genetic toolbox for manipulating Cm expands (51).

## MATERIALS AND METHODS

### Chlamydia strains.

Wild-type (WT) Cm (GenBank accession no. NC_002620) and Cm mutant GIAM-1 were propagated in HeLa 229 cells, and elementary bodies (EBs) were purified by discontinuous Renografin gradient centrifugation (52). GIAM-1 (previously referred to as delayed-development mutant) was isolated from an ethyl methanesulfonate-mutagenized C. muridarum library, and its *in vitro* growth characteristics have been described previously (30). For this study, GIAM-1 was purified by 3 rounds of plaque isolation in McCoy cells (53) and expanded in HeLa 229 cells, and EBs were purified as described above. Genomic DNA was extracted from purified EBs and sequenced as we described previously (54). Table 1 summarizes the missense and nonsense single nucleotide polymorphisms (SNPs) in GIAM-1 compared to the parent Cm wild-type strain.

### Mice.

Female C57BL/6 mice 6 to 8 weeks old were purchased from Jackson Laboratories (Bar Harbor, ME) and maintained in the animal facilities at the University of Arkansas for Medical Sciences (Little Rock, AR). All experimental procedures were performed in accordance with protocols approved by the UAMS Institutional Animal Care and Use Committee.

### Genital infection.

Female mice were challenged with WT Cm or GIAM-1 as described previously (28). Briefly, mice were injected subcutaneously with 2.5 mg of medroxyprogesterone acetate (Greenstone LLC, Peapack, NJ) 5 days prior to infectious challenge. Mice were then inoculated vaginally with 5 μl of sucrose-phosphate-glutamine (SPG) buffer (250 mM sucrose, 10 mM sodium phosphate, and 5 mM l-glutamic acid, pH 7.2) containing 5 × 10^4^ IFU of WT Cm or GIAM-1. To assess infection, vaginal swabs were collected on days 3, 7, 10, and 14 and weekly thereafter until infection was cleared, and infectious chlamydiae were enumerated as described below. Additionally, rectal swabs were collected weekly from day 28 to day 56 post-vaginal challenge to assess dissemination of chlamydiae to the gastrointestinal tract.

### Rectal infection.

Mice were restrained, the tip of a micropipette was inserted approximately 5 mm into the rectum, and 10 μl of SPG containing 1 × 10^5^ IFU of GIAM-1 or WT Cm was inoculated (28). Infection was monitored by collecting rectal swabs and enumerating infectious chlamydiae as described below. Additionally, vaginal swabs were collected to ensure that cross-inoculation from the gastrointestinal tract to the genital tract did not result from rectal challenge.

### Vaginal challenge of rectally infected mice.

Sixty days following rectal challenge with Cm or GIAM-1, mice were treated daily for 10 days with 300 μg of doxycycline by intraperitoneal injection to resolve chlamydia infection (28). Mice were rested for 2 weeks to allow clearance of residual antibiotic, and rectal swabs were collected for culture to confirm resolved infection. As described above for vaginal infection, mice were treated with medroxyprogesterone acetate and vaginally challenged with 5 × 10^4^ IFU of WT Cm, and infection was monitored by collecting vaginal swabs and enumerating infectious chlamydiae.

### Chlamydia cultures.

Vaginal and rectal swabs were placed into 1 ml SPG and processed as follows. Two 4-mm beads were added to the specimen and rotary shaken for 4 min to release chlamydia from the swabs. The swabs were removed, and samples were frozen at −80°C until processed. Enumeration of IFU has been detailed previously (55, 56).

### ELISA of sera and secretions.

Mice were bled prior to infection and at various time points during the course of infection. Serum was separated and stored at −20°C until analyzed. Vaginal secretions were collected by washing the vaginal vault twice with 80 μl of PBS containing 0.5% bovine serum albumin. Washes were immediately frozen at −80°C until analyzed. Antichlamydia antibody was measured by EB-ELISA as previously described, and titer was assigned as the highest dilution yielding an OD value of 0.250 or greater (56, 57).

### Statistical analysis.

GraphPad Prism 8 was used for data analysis. IFU data were analyzed by two-way analysis of variance (ANOVA) with Tukey’s multiple comparisons, and serum and vaginal wash antichlamydia antibody responses were compared by two-way ANOVA with Sidak’s multiple-comparison test.

### Data availability.

Genome sequence data sets associated with this study are available via GenBank under the accession numbers NC_002620 (Chlamydia muridarum parental wild type) and CP063055 (GIAM-1). All strains generated in the course of this study are available from the authors upon request.
